# Muscle-Specific Adaptations, Impaired Oxidative Capacity and Maintenance of Contractile Function Characterize Diet-Induced Obese Mouse Skeletal Muscle

**DOI:** 10.1371/journal.pone.0007293

**Published:** 2009-10-06

**Authors:** Karin E. Shortreed, Matthew P. Krause, Julianna H. Huang, Dili Dhanani, Jasmin Moradi, Rolando B. Ceddia, Thomas J. Hawke

**Affiliations:** 1 Department of Pathology & Molecular Medicine, McMaster University, Hamilton Ontario, Canada; 2 Muscle Health Research Centre, York University, Toronto, Ontario, Canada; 3 School of Kinesiology and Health Science, York University, Toronto, Ontario, Canada; Universidad Europea de Madrid, Spain

## Abstract

**Background:**

The effects of diet-induced obesity on skeletal muscle function are largely unknown, particularly as it relates to changes in oxidative metabolism and morphology.

**Principal Findings:**

Compared to control fed mice, mice fed a high fat diet (HFD; 60% kcal: fat) for 8 weeks displayed increased body mass and insulin resistance without overt fasting hyperglycemia (i.e. pre-diabetic). Histological analysis revealed a greater oxidative potential in the HFD gastrocnemius/plantaris (increased IIA, reduced IIB fiber-type percentages) and soleus (increased I, IIA cross-sectional areas) muscles, but no change in fiber type percentages in tibialis anterior muscles compared to controls. Intramyocellular lipid levels were significantly increased relative to control in HFD gastrocnemius/plantaris, but were similar to control values in the HFD soleus. Using a novel, single muscle fiber approach, impairments in complete palmitate and glucose oxidation (72.8±6.6% and 61.8±9.1% of control, respectively; p<0.05) with HFD were detected. These reductions were consistent with measures made using intact extensor digitorum longus and soleus muscles. Compared to controls, no difference in succinate dehydrogenase or citrate synthase enzyme activities were observed between groups in any muscle studied, however, short-chain fatty acyl CoA dehydrogenase (SCHAD) activity was elevated in the HFD soleus, but not tibialis anterior muscles. Despite these morphological and metabolic alterations, no significant difference in peak tetanic force or low-frequency fatigue rates were observed between groups.

**Conclusions:**

These findings indicate that HFD induces early adaptive responses that occur in a muscle-specific pattern, but are insufficient to prevent impairments in oxidative metabolism with continued high-fat feeding. Moreover, the morphological and metabolic changes which occur with 8 weeks of HFD do not significantly impact muscle contractile properties.

## Introduction

Sedentary behavior and consumption of high-energy diets favor the early development of disease states such as obesity, insulin resistance, and ultimately type 2 diabetes mellitus. These activities have created a serious health crisis in our society. In the United States alone, it is estimated that approximately 57 million people have pre-diabetes (impaired glucose tolerance preceding type 2 diabetes mellitus development). Of this, over 2 million are under the age of 20 years old; an age group that until recently was generally unaffected by these disorders [1].

As skeletal muscle plays a major role in energy expenditure and insulin-stimulated glucose disposal, understanding changes that occur to this tissue with obesity and pre-diabetes development are critical to elucidating the underlying causes for insulin resistance and type 2 diabetes. Though a number of studies have investigated the effects diet-induced obesity on skeletal muscle oxidative capacity and insulin sensitivity, we are unaware of any that relate these changes with alterations in skeletal muscle morphology and functional capacity. Undertaking a comprehensive analysis in a variety of muscles is particularly important given that the disparities in model used, length and composition of diet intervention, muscles analyzed and analytical techniques have made comparisons between studies very challenging. For example, an increased [2], decreased [3]–[5] or unchanged [4] capacity for oxidative metabolism with high fat diet intervention have all been demonstrated. Furthermore, an increase in oxidative phosphorylation protein complexes have been measured in the gastrocnemius muscles following 4 weeks of high fat feeding [6], while a decreased expression of oxidative phosphorylation genes and cytochrome C protein has been demonstrated in the quadriceps muscles in response to 3 weeks of high fat diet consumption [7]. It is worth noting that studies assessing oxidative metabolism have investigated changes through the utilization of homogenized muscle, skinned fibers and/or isolated mitochondria. While these techniques are valuable tools for the assessment of specific aspects of mitochondrial oxidative capacity, the disruption of the muscle may eliminate potential impairments in fatty acid uptake, transport, and trafficking inside the cell caused by high fat diet, precluding extrapolation of this data to the intact whole muscle.

Thus, it was the aim of the current study to comprehensively analyze skeletal muscle morphology, metabolism and contractile function in young adult skeletal muscle. Specifically, we assessed skeletal muscle glucose and fatty acid oxidation rates in isolated muscle fibers and in intact oxidative and mixed muscles along with key oxidative enzyme activities, skeletal muscle morphology, and contractile properties in mice fed either a high fat diet (HFD) or standard rodent chow (control) for 8 weeks. Our findings indicate that the skeletal muscle of mice with diet-induced obesity undergoes significant alterations in fiber type, fiber area and intramyocellular lipid (IMCL) levels and that these changes occur in a muscle-specific manner. However, complete glucose and palmitate oxidation rates were decreased in all muscles analyzed, suggesting that elevated IMCL levels and alterations in SCHAD activity do not solely explain the insulin resistance and impaired oxidative capacities. Moreover, peak tetanic force and overall fatigue rates were maintained despite significant changes in muscle morphology and oxidative capacity.

## Results

### High fat diet induces insulin resistance and excessive weight gain in young adult mice

**Figure 1:**
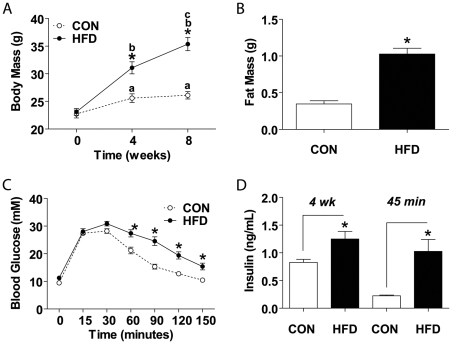
Eight weeks of a high fat diet (HFD) elicits pre-diabetes. (A) Fasted body mass was assessed before experimental diet began and after 4 and 8 weeks (N = 19 CON, 20 HFD). (B) Epididymal fat mass after 8 weeks of diet intervention (N = 19 CON, N = 20 HFD). (C) Intraperitoneal glucose tolerance test (IPGTT) performed after an overnight fast (16 hrs) 1 week before harvest (N = 19 CON, N = 18 HFD). (D) Plasma insulin levels assessed 4 weeks into diet intervention (8 hr fast, N = 10) and at IPGTT 45 minute time-point (16 hr fast, N = 4). Significance is represented by * vs. CON at same time point (A–D), a or b vs. 0 weeks within diet group, and c vs. 4 weeks within diet group, p<0.005.

Young adult mice fed a standard chow diet continued to gain weight during the first 4 weeks of experimental assessment, verifying that these mice were still within the growth and maturation phase of development (Figure 1A). Mice consuming a diet with 60% kcal from fat (HFD) are significantly heavier than the standard chow fed controls as early as 4 weeks from the onset of HFD. After 4 weeks of diet intervention, the body weight of control mice stabilized, whereas HFD mice continued to increase, such that by the time of harvest, there was nearly a 40% gain in body mass (Figure 1A).

There was an approximate 2.5-fold increase in epididymal fat mass (Figure 1B) without any differences in absolute tibialis anterior (TA) or soleus muscle masses between groups (Control TA: 52.2±1.4 mg, Control soleus: 7.7±0.7 mg; HFD TA: 51.7±1.4 mg, HFD soleus: 7.7±0.5 mg).

There was no difference in fed blood glucose levels after 6 weeks of diet intervention (Control: 10.2±0.3 mM; HFD: 11.6±0.7 mM) or fasted blood glucose levels after 7 weeks of diet intervention (Figure 1C, time 0). However HFD mice displayed a significantly reduced capacity for glucose clearance in response to an intraperitoneal glucose tolerance test (IPGTT), indicating insulin resistance (Figure 1C). Insulin resistance was further demonstrated by elevated resting plasma insulin levels at 4 weeks of diet intervention and at the 45 minute time point of the IPGTT in HFD mice (Figure 1D).

### HFD results in a shift towards more and larger oxidative fibers

**Figure 2:**
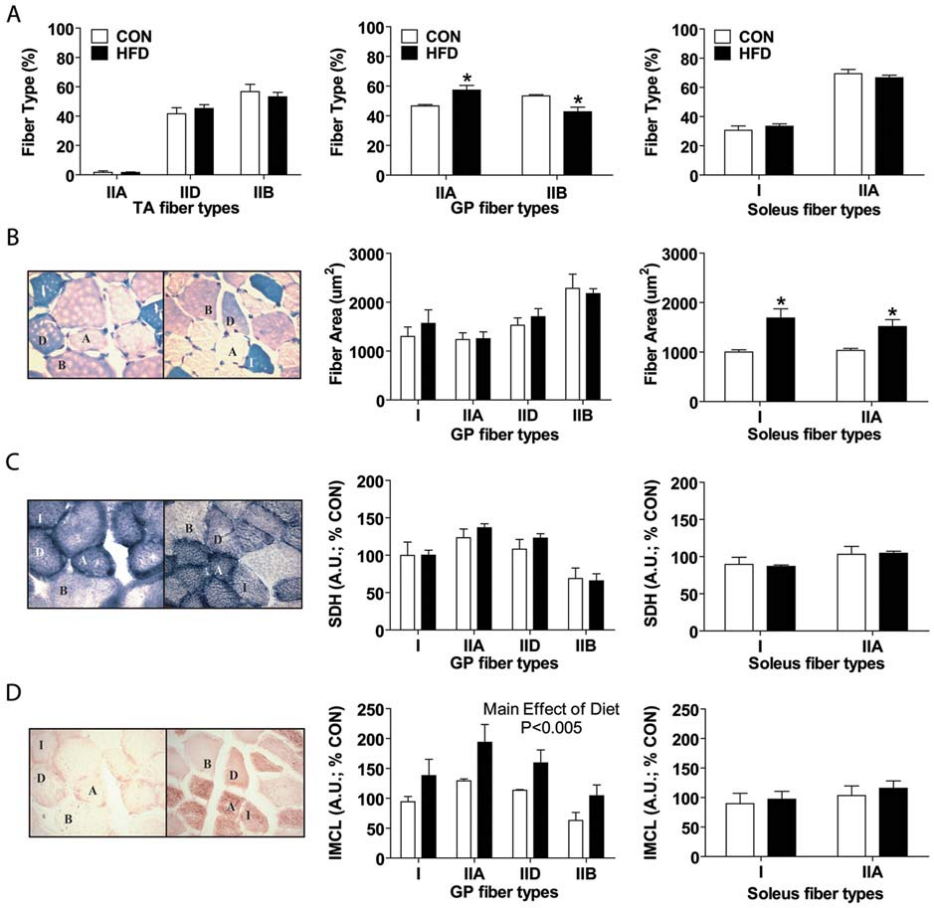
Morphometric changes are muscle-specific. Serial cross-sections from control (CON, white bars in all graphs) and high fat diet (HFD, black bars in all graphs) fed mouse muscles [TA (top left only), gastrocnemius/plantaris complex (GP, all middle graphs), and soleus (all right graphs)] were examined for (A) fiber type composition, (B) area, (C) SDH stain intensity, and (D) IMCL stain intensity. Representative images of all stains used, performed on GP muscle cross-sections are shown to the left of graphs B–D with fiber type (type I, and types II- A, D, B) labeled with CON on the left and HFD on the right. (B) Metachromatic fiber type stain was used to assess fiber type. (C) SDH and (D) Oil-Red-O stains are graphically represented by arbitrary units (A.U.) of optical intensity measurements, with greater values for more intense stains and normalized to a percentage of all the control value means for each graph (% CON). All measurements were taken on an average of 51–331 total fibers/animal with a Nikon Eclipse 90i microscope (N = 3–4). Significance is represented by * vs. CON, p<0.005.

In HFD gastrocnemius/plantaris muscles, type I and IIA fiber type proportions increased (IIA: 40.3±3.5% vs. 32.9±1.9%, I: 9.80±1.8% vs. 5.5±1.8%, HFD vs. control, respectively), while type IID and IIB fiber types were decreased in number (IID: 20.2±2.1% vs. 23.8±3.9%, IIB: 29.7±1.5% vs. 37.7±2.7%, HFD vs. control, respectively; main effect of interaction, p<0.05). Considering that type IIA and IIB fibers represent the most oxidative and glycolytic fiber types in mouse skeletal muscle respectively [8], and that these two fiber types comprise more than 70% of all fibers we quantified in gastrocnemius/plantaris muscles, we specifically compared the change in fiber type percentages between these two fiber types (Figure 2A middle panel). HFD caused a significant rise in the percentage of oxidative IIA fibers and a significant decrease in the percentage of glycolytic IIB fibers in the gastrocnemius/plantaris muscles. In the TA and soleus muscles, no change in fiber type percentages were observed between HFD and control groups (Figure 2A left and right panels).

Assessment of cross-sectional area per fiber type revealed no differences in the gastrocnemius/plantaris muscles (Figure 2B middle panel) between groups. However a significant increase in the cross-sectional area of both type I and IIA muscle fibers (which comprise the entire muscle) was observed in HFD soleus compared to control (Figure 2B right panel).

Histological staining for succinate dehydrogenase (SDH) activity in the control muscle was greatest in fibers identified by metachromatic stain as type IIA followed by IID≥I>IIB, respectively. This finding validated the fiber type (metachromatic) staining results. No significant differences in SDH enzyme activity were detected between HFD and control in either the gastrocnemius/plantaris or soleus muscles, regardless of fiber-type (Figure 2C middle and right panel).

As expected, the IMCL levels across fiber types were different; with oxidative fiber types displaying greater absolute IMCL levels than glycolytic fiber types. HFD gastrocnemius/plantaris muscles displayed a significant increase in IMCL levels compared to control muscles (Figure 2D middle panel). All fiber types within the HFD gastrocnemius/plantaris displayed a similar percent increase (∼50–60%) in IMCL levels above that measured in the respective control fiber type. Interestingly IMCL levels relative to cross-sectional area in the soleus muscles were not different between control and HFD regardless of fiber type (Figure 2D right panel). The observed increase in HFD soleus muscle cross-sectional area may have allowed for dispersion of IMCLs such that the density of IMCLs was unchanged between diet groups.

### Complete palmitate and glucose oxidation rates are impaired in HFD skeletal muscle

**Figure 3:**
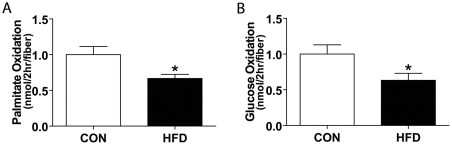
Impaired palmitate and glucose oxidation in HFD single fibers. (A) Palmitate (N = 19, average of 17 fibers/dish) and (B) glucose (N = 12 CON, N = 10 HFD, average of 23 fibers/dish) oxidation in single fibers derived from EDL and peroneus muscles was similarly impaired in mice fed a high-fat diet (HFD) compared to control (CON). Values were normalized to control values for each experiment and significance is represented by * vs. CON, p<0.005.

Complete glucose and fat oxidation (to CO_2_) were assessed using a novel, intact single muscle fiber protocol, as well as the well-established whole skeletal muscle preparation [9], [10]. The whole muscle preparation was used to confirm the palmitate oxidation results in single fibers and by assessing glucose oxidation and glycogen synthesis we could further demonstrate insulin resistance within the intact skeletal muscle of HFD mice. Palmitate oxidation rates in intact fibers from extensor digitorum longus (EDL) and peroneus longus muscles of the HFD were approximately 65% of that measured in control muscle fibers (Figure 3A). Using this single fiber technique, we also demonstrated that basal glucose oxidation rates were significantly diminished in single fibers from HFD muscle compared to single fibers from control fed mice (Figure 3B).

**Figure 4:**
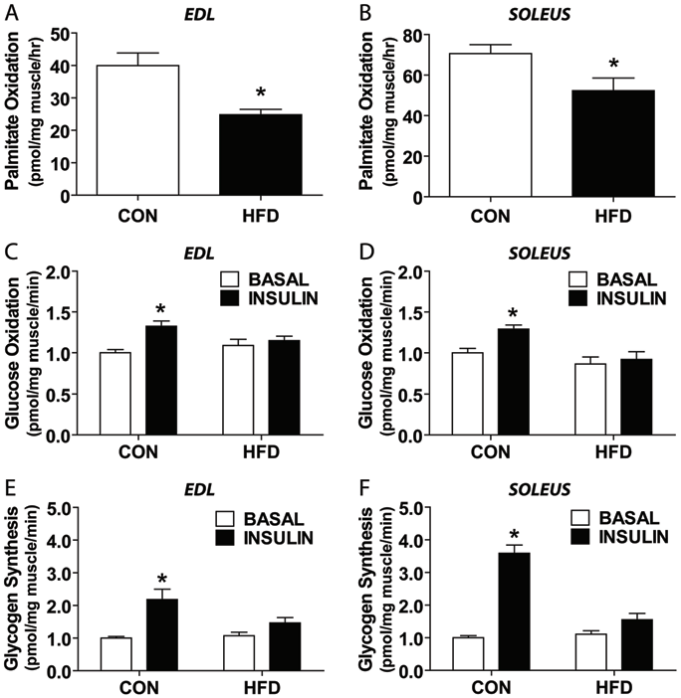
Impaired oxidation, glycogen synthesis, and insulin stimulated response in HFD muscle. Palmitate oxidation in whole (A) EDL (N = 4) and (B) soleus (N = 4) muscles is impaired with HFD. Both glucose oxidation in whole (C) EDL and (D) soleus and glycogen synthesis in whole (E) EDL and (F) soleus muscles with HFD demonstrate a significant blunted response to insulin pre-incubation (INSULIN), vs. no insulin pre-incubation (BASAL), in HFD muscle (N = 4–6, two-way ANOVA with Bonferonni post-tests between insulin conditions within diet). Significance is represented by * vs. CON (A–B), and vs. INSULN (C–F), p<0.005.

Similar results to those acquired in intact single fibers were obtained when intact EDL and soleus muscles were used to assess palmitate (Figure 4A–B). While whole body insulin resistance was demonstrated by IPGTT and elevated insulin levels at rest and in response to IPGTT, we were interested in validating skeletal muscle insulin resistance and determining if there were muscle-specific differences in insulin resistance in HFD mice. Using intact soleus and EDL muscles, we undertook insulin stimulated glucose oxidation and glycogen synthesis assays (N = 4–6 muscles per group). A consistent blunting of the insulin-stimulated response was observed in all HFD muscles (Figure 4C–F). Importantly, this observation is made despite the soleus being a purely oxidative muscle (type I and IIA) and the EDL being a mixture of glycolytic (type IIB) and oxidative (type IID) fiber types. Note that synthesis rates were higher in soleus compared to EDL muscles as would be expected based on their fiber type distribution [11,present study].

### Metabolic enzyme activities in HFD and control muscles

**Figure 5:**
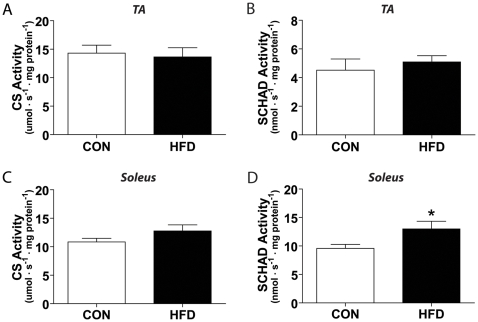
Oxidative enzyme alterations are muscle specific. Citrate synthase (CS) activity is unaltered in both (A) TA and (C) soleus muscle between control (CON) and high fat diet (HFD). Short chain 3-β-hydroxyacyl coenzyme-A dehydrogenase (SCHAD) activity is unaltered in (B) TA muscle, though (D) soleus muscle from HFD mice exhibits SCHAD activity 136% of CON. Significance is represented by * vs. CON, p<0.005.

Citrate synthase (CS) and SCHAD are key enzymes in the TCA (tricarboxylic acid) cycle and β-oxidation pathways, respectively. No difference between HFD and control muscle CS or SCHAD activity was observed in homogenates from the TA muscles (Figure 5A–B). In homogenates from the soleus muscles, CS activity was not different between groups, though a significant increase in SCHAD enzyme activity was observed with HFD compared to control (Figure 5C–D).

As mentioned previously, an SDH activity stain was undertaken. In fibers identified as type IIB by metachromatic staining, SDH staining was of the lowest intensity, while fibers identified as type IIA by metachromatic staining displayed the most intense SDH staining. Thus, SDH activity staining validated the fiber type data obtained by metachromatic staining. This analysis also allowed us to determine if fiber type specific adaptations to SDH enzymatic activity and mitochondrial content were occurring in HFD versus control muscle. No differences in mean SDH staining intensity were observed between HFD and control muscles in the soleus or gastrocnemius/plantaris muscles, regardless of fiber type (Figure 2C).

### Muscle contractility is not significantly altered following 8 weeks of high-fat diet

**Figure 6:**
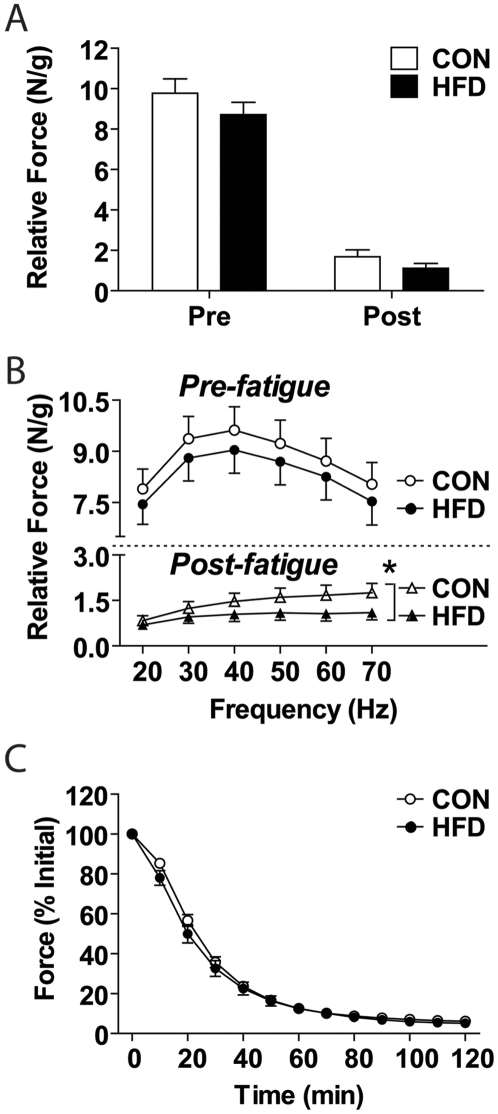
*In situ* contractile analysis reveals trend towards force decrements, yet unaltered peak force and fatigue. Relative tetanic force production [in Newtons (N) per gram (g) of wet muscle mass] in the gastrocnemius/plantaris muscle group of high-fat diet (HFD) mice compared to control (CON) was (A) not different before (Pre) or after (Post) the fatigue protocol. (B) There was no difference between diets over all frequencies used to test force production pre-fatigue, however there was a significant main effect of diet post-fatigue. (C) Contractile force, relative to initial (% initial), throughout a 2 minute low-frequency fatigue protocol was not different between diet groups. Significance is represented by * vs. CON, p<0.005.

The peak tetanic force pre- and post-fatigue was not significantly different between control and HFD (Figure 6A). When the pre-fatigue force-frequency curve was plotted, an insignificant, though consistently lower force production at each stimulation frequency was observed in the HFD fed mouse compared with control (main effect of diet: P = 0.089; Figure 6B top panel). Analysis of the post-fatigue force-frequency curve revealed that the ability to immediately recover force following a low-frequency fatigue protocol was impaired in HFD muscle compared with control (main effect of diet: P<0.05; Figure 6B bottom panel). Though a significant level of fatigue was induced in both control and HFD groups, there was no difference between groups in rate or degree of fatigue development (Figure 6C).

## Discussion

Here we provide novel evidence that the skeletal muscles of mice fed a high fat diet for 8 weeks develop pre-diabetes, undergo muscle-specific morphological and enzymatic adaptations and exhibit impairments in complete glucose and fat oxidation. These changes in muscle morphology and metabolism did not have significant effects on peak tetanic force and low-frequency fatigue rates. These results demonstrate for the first time that skeletal muscle health is impaired in the pre-diabetic state and that despite these significant impairments in oxidative metabolism, there is a maintenance of muscle contractile properties.

A significant muscle-specific shift towards a greater contribution of oxidative fibers was found in mice fed a HFD. In fact, the gastrocnemius/plantaris muscles increased the percentage of oxidative fibers, while the soleus increased fiber cross-sectional area and the TA displayed no fiber type change. This is in line with a recent study reporting that HFD led to increased soleus muscle fiber areas, but not fiber type proportions and no change in fiber type percentage or area in the EDL [12]. The shift in oxidative potential of the soleus and gastrocnemius/plantaris, but not the TA, may be the result of an increased activity in the calf musculature compared to dorsiflexors in relatively sedentary rodents. The shift towards a greater oxidative fiber contribution seen in the present study is also consistent with that observed by others [6], [13], [14]. Of note, deWilde et al. [6] found that following 3 and 28 days of high-fat feeding (45% kcal from fat) there was a significant increase in the mRNA and protein expression of slow myosin heavy chain and other markers of the slow oxidative fiber phenotype. While myosin ATPase expression, demonstrated using metachromatic staining, is often correlated with oxidative potential of the muscle, a shift in myosin ATPase expression does not directly indicate there is an increase in oxidative potential. Here we substantiate the shift towards an increased oxidative potential using SDH activity staining. The SDH enzyme catalyzes the conversion of succinate to fumarate in the TCA cycle. It consists of two large subunits which form complex II of the mitochondrial respiratory chain along with two smaller subunits, which attach SDH to the inner mitochondrial membrane. SDH staining has been shown to be extremely useful for detecting variations in the fiber distribution of mitochondria, particularly in states of mitochondrial dysfunction [15], [16].

In our experiments, the mean intensity of SDH staining per fiber type was not different between control and HFD muscles, although soleus muscle had increased fiber cross-sectional area (Figure 2D). If increased fiber areas or a shift towards more oxidative fibers were not associated with a concomitant increase in mitochondrial content, then the mean pixel intensity for SDH would have decreased proportionally. However, since there was a proportional increase in SDH density in our study, we interpret that a relative increase in mitochondrial content has occurred in HFD muscles, indicating a shift towards an increased oxidative potential. This could represent an early positive adaptation to the abundance of lipids and elevated IMCL levels typical of the HFD.

In fact, IMCL levels were increased with HFD in this study, albeit in a fiber type and muscle-group specific manner. There were a number of ways in which an increased IMCL deposition was observed in this study including greater IMCL density (observed in the gastrocnemius/plantaris), increased oxidative muscle fiber area with unchanged IMCL density (observed in the soleus), and increased oxidative muscle fiber percentages (observed in the gastrocnemius/plantaris). Although our findings suggest an early attempt to enhance oxidation in response to high-fat feeding, after 8 weeks of such a diet a downward spiral seemed to develop leading to impaired glucose and lipid oxidation rates. This is consistent with previous observations showing time-dependent alterations in mitochondrial density or structure within the gastrocnemius muscles of mice fed a high-fat/high-sugar (HFHSD) diet [17]. In fact, Bonnard and colleagues [17] found that significant alterations in mitochondrial density or structure occurred only after 16 weeks of HFHSD. It was further reported that 4 weeks of diet intervention was associated with some metabolic perturbations that could reflect the initiation of deleterious processes in pre-diabetic skeletal muscle. Our study also provides evidence of early adaptive responses to HFD, which are followed by impairment in complete glucose and lipid oxidation. Furthermore, it is important to note that the changes in muscle morphology and metabolic enzyme activities are occurring in a muscle-specific and time-dependent manner. Therefore, caution is warranted when deriving conclusions based on the analyses of one specific muscle or limited metabolic markers.

In an attempt to address the apparent discrepancy between a shift towards an oxidative muscle phenotype and the reduced lipid and glucose oxidation rates, we assessed the activity of CS and SCHAD in muscles that either demonstrated an increase (soleus) or no change (TA) in oxidative potential in response to HFD. We did not find any significant differences in CS and SCHAD activities in the TA muscles of control and HFD mice. These results were consistent with the lack of change in fiber type proportion in this muscle. Interestingly, we found a significant increase in SCHAD, but not in CS activity, in the HFD soleus. Whether the increase in SCHAD activity in the HFD soleus is the result of the increased cross-sectional area and proportionate increase in mitochondrial content remains to be determined. However, since oxidative muscle fiber types increased in size (soleus) or number (gastrocnemius/plantaris) and a concomitant rise in SDH staining intensity was found, it suggests that 8 weeks of HFD did not decrease mitochondrial content. In fact, we would argue that the uniform increase in staining throughout the fibers is indicative of an increase in SDH content resultant from an overall increase in mitochondrial content and not solely increased SDH activity. Importantly, regardless of whether there was an increase in mitochondrial content or a fiber type specific increase in oxidative potential of muscles exposed to HFD, a significant impairment in complete glucose and palmitate oxidation was detected in our studies. These observations were made using a novel isolated single fiber technique and validated with the classical, isolated muscle methodology [9], [10]. The isolated single fiber approach facilitates exchange of substrates and gases with the incubation medium and maintains all populations of mitochondria (subsarcolemmal and intermyofibrillar). Furthermore, it preserves the mitochondria and metabolic enzymes in their natural surrounding. Therefore, both approaches allowed us to study the ability of both the skeletal muscle cell and the skeletal muscle tissue from mice fed a HFD to metabolize glucose and fatty acids.

A number of studies have demonstrated that a prolonged exposure to elevated levels of fatty acids lead to insulin resistance and impair skeletal muscle glucose and lipid metabolism. The buildup of harmful lipid metabolites including long-chain fatty acyl CoA, diacylglycerol, and ceramides have been proposed to cause these deleterious metabolic effects in skeletal muscle [18]–[20]. Furthermore, a recent study using rats fed a HFD (45% fat for 12 weeks) demonstrated an increase in incomplete β-oxidation with excess acid-soluble metabolites being produced, indicating depletion of intermediates of the TCA cycle [4]. These authors also demonstrated the importance of fatty acid entry into the mitochondria, and not simply increased IMCL, to elicit HFD induced insulin resistance [4]. In this context, our findings that the increase in SCHAD activity was not followed by a proportional increase in CS activity suggest a flux through β-oxidation that is not proportionally matched by the TCA cycle. Furthermore, the fact that soleus muscles displayed insulin resistance and impaired complete oxidation of lipids and glucose even in the face of elevated SCHAD activity and normal IMCL levels, support the idea that HFD causes insulin resistance through lipotoxicity specifically within mitochondria [4], [21].

Despite significant alterations in muscle morphology and metabolism, no significant impairment in peak tetanic force either pre- or post-fatigue was observed. However when we analyzed the force-frequency curves, we see that at all stimulation frequencies tested, HFD skeletal muscle consistently displayed reduced force production regardless of stimulation frequency in the pre-fatigue state (P = 0.089). We hypothesize that the early adaptations that occurred in response to HFD attenuated muscle force loss and continued exposure to a HFD would ultimately result in significant decreases in contractile force, consistent with that observed in humans [22]. The absence of a difference between groups in response to a low-frequency fatigue protocol is likely explained by the low intensity and short duration of this protocol. That is, this submaximal fatigue protocol was insufficient to elicit differences due to changes in oxidative metabolism. Though no change in peak tetanic force post-fatigue was observed, analysis of the force-frequency curve revealed a significant main effect of diet (Figure 6B). This finding would imply that following fatiguing contractions, the ability to immediately recover force is attenuated in HFD muscles compared to control muscles. Given the impaired metabolic capacity in the HFD muscles, this result was not unexpected. While it would be premature to extrapolate too much from this result, we hypothesize that the ability to respond to exercise or exercise- training may be impaired in these pre-diabetic mice.

Overall, the current study provides, for the first time, a comprehensive analysis of skeletal muscle morphology, metabolism and function following 8 weeks of high fat diet consumption. Our findings substantiate the proposal of deWilde and colleagues [6] that skeletal muscle responds to high fat diet intervention with an early, positive adaptive response. However, with continued high-fat diet exposure, perturbations in gene and protein expression will ultimately result, causing decreased oxidative capacity at a later stage. Taken together, this work advances our understanding of skeletal muscle health prior to the development of type 2 diabetes mellitus and, in part, aids in explaining the variability that has been observed in previous studies investigating pre-diabetic skeletal muscle. Furthermore, these results support the undertaking of early therapeutic interventions in obese, pre-diabetic youth prior to significant long-term effects on muscle growth and function.

## Materials and Methods

### Animals and blood sampling

All experimental protocols were approved by the York University Animal Care Committee in accordance with Canadian Council for Animal Care guidelines.

Male C57BL/6J mice were obtained from Jackson Laboratories (Bar Harbor, ME). Animals were housed in temperature and humidity-controlled facility with a 12/12 h light/dark cycle and had *ad libitum* access to water and food. After an initial acclimatization, animals (10 weeks of age; N = 20 per group) were randomly assigned to either a high fat diet [HFD; TestDiet, cat#58126: energy (kcal/g) from protein (18.3%), fat (60.9%), carbohydrate (20.1%)] or standard mouse chow [LabDiet 5015 Mouse Diet: energy (kcal/g) from protein (20%), fat (25%), carbohydrate (55%)]. Body mass and blood glucose (via tail nick; OneTouch Ultra glucometer; Johnson & Johnson) were assessed in the fed state on biweekly basis. Fasted body weight was assessed at 4 weeks (following an 8 hr fast) and 8 weeks (following a 16 hr fast) of diet intervention. Fasted (8 hr) plasma insulin was assessed at 4 weeks of diet intervention as described below for insulin from the intraperitoneal glucose tolerance test (IPGTT).

An IPGTT was performed on mice fasted overnight (16 hrs) after 7 weeks of diet intervention. Glucose was injected IP (2 g/kg of body weight) and blood glucose was assessed by tail bleeds at 0, 15, 30, 60, 90, 120, 150 min. Plasma was collected by tail bleed at 45 min and later analyzed for insulin, in duplicate, using 5 µl in the Ultra Sensitive Mouse Insulin ELISA Kit (cat#90080, Crystal Chem, Illinois), according to the manufacturer's instructions.

### Experimental procedures

Following 8 weeks of diet, mice were fasted overnight (16 hrs) and weighed. Subsequently, mice were anaesthetized, the muscle stimulation protocol (described below) was performed and tissues were harvested. Immediately prior to the electrical stimulation protocol, the left and right tibialis anterior (TA), extensor digitorum longus (EDL), peroneus, and soleus muscles were removed from the muscle-stimulation leg (left). EDL and peroneus longus muscles were used for single fiber isolation and soleus was used for intact muscle oxidative capacity, snap frozen or mounted with tissue freezing medium and frozen in isopentane cooled by liquid nitrogen. The TA muscles were snap frozen for future analysis or mounted with tissue freezing medium and frozen in isopentane cooled by liquid nitrogen. Following electrical stimulation, the gastrocnemius/plantaris complex was weighed and then either mounted with tissue freezing medium and frozen in isopentane cooled by liquid nitrogen for subsequent histological analysis or snap frozen in liquid nitrogen for future analysis.

### Muscle stimulation protocol

Prior to surgery, mice were injected with ketamine/xylazine (150 mg/kg: 10 mg/kg) and the left gastrocnemius/plantaris complex was isolated from its distal insertion, attached to a force transducer and optimal voltage and length were determined [23], [24]. A force-frequency curve was determined before (pre-) and after (post-) a fatigue protocol consisting of 2 minutes of low-frequency (30 Hz) stimulations lasting 333 ms in 1 s trains. Pre-fatigue force determination consisted of 1 s stimulation every 30 s at increasing frequencies of 10 Hz starting at 20 Hz [23], [24]. Post-fatigue force determination was similarly conducted except that stimulations were spaced 10 s apart, instead of 30 s, in order to ensure that peak force determination was completed prior to recovery from the fatigue protocol as determined by pilot studies. The post-fatigue force-frequency curve provided an assessment of immediate force recovery following the fatigue protocol. All muscle function data were collected through an AD Instruments Bridge Amp and Powerlab 4/30, and analyzed with Chart5 PowerLab software.

### Single muscle fiber isolation

Single skeletal muscle fibers were harvested from the EDL and peroneus longus muscles using a collagenase digestion protocol as previously described [25]. Single fibers were collected using glass blown Pasteur pipettes and placed in matrigel-coated 35 mm culture dishes (BD Biosciences, Canada) containing plating media [10% horse serum (Invitrogen, USA), 0.5% chick embryo extract (MP Biomedicals, Ohio) in Dulbecco's Modified Essential Medium (DMEM; Invitrogen, USA)].

### Fatty acid and glucose oxidation in single fibers

Approximately 18 hours following isolation, palmitate and glucose oxidation rates were assessed on groups of single fibers in 35 mm cell culture dishes. The 18 hour incubation period was critical to allow fibers to recover from the isolation procedure, settle onto the matrigel-coated dishes and allow any non-viable fibers to hypercontract and hence be removed with rinses. After the incubation period, wells were slowly rinsed three times with warm PBS and viable fibers were counted. On average, 15 to 30 viable, healthy fibers from each mouse muscle were used for oxidation rate assessment. Fiber viability was assessed by the presence of cross-striations along the length of fiber and the absence of sarcolemmal damage. Fibers were then serum-starved for 3 hours in low-glucose DMEM supplemented with sodium bicarbonate and pH adjusted. Following starvation fibers were incubated with either [U-^14^C]glucose (0.2 µCi/ml) + cold glucose (5.5 mM) for the determination of glucose oxidation or [1-^14^C]palmitic acid (0.15 µCi/ml) + cold palmitate (100 µM) complexed with fat-free BSA + L-carnitine (500 µM) for the determination of palmitate oxidation. Each well was incubated with the palmitate or glucose mixture for 2 hours in a closed system then the CO_2_ produced was assessed as previously described [9], [10].

### Whole muscle glucose and fatty acid oxidation and glycogen synthesis

Following 8 weeks of diet intervention, mice were fasted overnight and anesthetized with ketamine/xylazine (150 mg/kg: 10 mg/kg) prior to removal of soleus and EDL muscles. Palmitate oxidation (N = 4 per muscle group), glucose oxidation (N = 4–6 per muscle group), and glycogen synthesis (N = 4–6 per muscle group) were assessed as previously described [9], [26], [27]. Briefly, isolated EDL and soleus muscles were quickly extracted and mounted onto thin, stainless steel wire clips to maintain resting length. The incubations were performed immediately after extraction and the muscles were placed in plastic scintillation vials containing 2 ml of gassed (O_2_∶CO_2_ – 95∶5%) Krebs-Hanseleit bicarbonate (KHB) buffer with 4% fat-free BSA and 5.5 mM glucose. The scintillation vials were then sealed with rubber stoppers and gasification (O_2_∶CO_2_ – 95∶5%) was continued during all incubations. After pre-incubation, the muscles were transferred to a second set of vials with 1.5 ml of KHB buffer containing either [U-^14^C]glucose (0.2 µCi/ml) + cold glucose (5.5 mM) for the determination of glucose oxidation and glycogen synthesis or [1-^14^C]palmitic acid (0.15 µCi/ml) + cold palmitate (0.2 mM) complexed with fat-free BSA + L-carnitine (500 µM) for the determination of palmitate oxidation. Assessment of the effects of insulin on glucose oxidation and glycogen synthesis was performed in the presence of 100 nM of the hormone.

### Enzymatic assays

Determination of citrate synthase (CS) and short chain fatty acyl CoA dehydrogenase (SCHAD) was performed using pulverized TA (∼25 mg) or soleus (∼5 mg) muscle powder sonicated with 1∶20 (w/v) of extraction buffer as previously described [28], assayed using a spectrophotometer (BioRad SmartSpecPlus, CA) and normalized to protein concentration determined by Bradford assay [28]. CS activity was assessed in duplicate or triplicate and measurements were taken every 10 s over a 3 min period. SCHAD activity was assessed using both 5 µl and 10 µl samples to determine optimal volume, measurements were taken every 2 s over a 2.5 min period.

### Histochemical analyses

Fiber type and IMCL were assessed using the metachromatic and Oil-Red-O staining methods respectively [24], [25], [29], [30]. Metachromatic stained muscle sections were used to assess fiber type areas for gastrocnemius/plantaris (N = 4, average 187 fibers/muscle) and soleus (N = 3, average 214 fibers/muscle) and fiber type percentages for gastrocnemius/plantaris (N = 4, average 187 fibers/muscle), soleus (N = 3, average 328 fibers/muscle), and TA (N = 4, average 331 fibers/muscle) using Scion Image. Succinate dehydrogenase (SDH) activity was assayed by incubation of muscle sections in medium consisting of 100 mM phosphate buffer (pH 7.6), 1 mM KCN (Sigma, 207810), 6.3 mM EDTA, and 1.22 mM nitroblue tetrazolium (Sigma, N6876). IMCL and SDH intensity were quantified in representative mixed fiber type regions in serial sections of the gastrocnemius/plantaris (IMCL: N = 4 average 187 fibers/muscle; SDH: N = 4, average 187 fibers/muscle) and soleus (IMCL: N = 4–5, average 51 fibers/muscle; SDH: N = 3, average 62 fibers/muscle). To measure SDH activity per fiber, images were converted to grey scale, fibers were encircled in Adobe Photoshop and the mean pixel intensity/optical intensity, graphically represented as arbitrary units (A.U.) relevant to the overall control mean, in the area of interest was recorded. Consequently, the darker the stain per fiber, the more SDH activity, the greater the mean pixel intensity. The same procedure was undertaken to assess IMCL levels using Oil-Red-O staining with the increase in lipid droplets resulting in a greater red color and thus a greater value for mean pixel intensity. All images were acquired with a Nikon Eclipse 90*i* microscope and Q-Imaging MicroPublisher 3.3 RTV camera with Q-Capture software. All control and HFD images for each morphometric analysis were taken at the same exposure with the same microscope settings.

### Data analyses

All statistical analyses were performed with GraphPad Prism 5 software. Differences between groups were determined using the appropriate student t-test, one-way or two-way ANOVA followed by Bonferonni post-hoc tests when appropriate. P values less than 0.05 were considered significant. All data presented are mean±standard error of the mean.
